# Surgical safety and effectiveness of bilateral axillo-breast approach robotic thyroidectomy: a systematic review and meta-analysis

**DOI:** 10.1016/j.bjorl.2023.101376

**Published:** 2023-12-15

**Authors:** Yun Jin Kang, Gulnaz Stybayeva, Se Hwan Hwang

**Affiliations:** a Department of Otorhinolaryngology-Head and Neck Surgery, Soonchunhyang University College of Medicine, Cheonan, Republic of Korea; b Department of Physiology and Biomedical Engineering, Mayo Clinic, Rochester, United States; c Department of Otolaryngology-Head and Neck Surgery, The Catholic University of Korea, College of Medicine, Bucheon St. Mary’s Hospital, Seoul, Republic of Korea

**Keywords:** Surgery, robot-assisted, Robotic surgical procedures, Thyroidectomy, Thyroid neoplasms, Thyroid nodule

## Abstract

**Objectives:**

To assess the safety and effectiveness of bilateral axillo-breast approach robotic thyroidectomy in thyroid tumor.

**Methods:**

Bilateral axillo-breast approach robotic thyroidectomy and other approaches (open thyroidectomy, transoral robotic thyroidectomy, and bilateral axillo-breast approach endoscopic thyroidectomy) were compared in studies from 6 databases.

**Results:**

Twenty-two studies (8830 individuals) were included. Bilateral axillo-breast approach robotic thyroidectomy had longer operation time, greater cosmetic satisfaction, and reduced transient hypoparathyroidism than conventional open thyroidectomy. Compared to bilateral axillo-breast approach endoscopic thyroidectomy, bilateral axillo-breast approach robotic thyroidectomy had greater amount of drainage, lower chances of transient vocal cord palsy and permanent hypothyroidism, and better surgical completeness (postopertive thyroblobulin level and lymph node removal). Bilateral axillo-breast approach robotic thyroidectomy induced greater postoperative drainage and greater patient dissatisfaction than transoral robotic thyroidectomy.

**Conclusion:**

Bilateral axillo-breast approach robotic thyroidectomy is inferior to transoral robotic thyroidectomy in drainage and cosmetic satisfaction but superior to bilateral axillo-breast approach endoscopic thyroidectomy in surgical performance. Its operation time is longer, but its cosmetic satisfaction is higher than open thyroidectomy.

## Highlights

• Bilateral Axillo-Breast Approach Robotic Thyroidectomy (BABART) has been developed.
• We directly compare BABART with other approaches in outcomes and complications.
• BABART's operation time, drainage, and cosmetic satisfaction were insufficient.
• However, the surgical performance of BABART was not inferior.

## Introduction

According to literature, thyroid surgery was performed using a knife for the first time in 1646, but the mortality rate was high.^1^ Theodor Kocher reported that the mortality rate after thyroid surgery was reduced to 0.2% in 1898, and conventional Open Thyroidectomy (OT) also made many advances in reducing complications.^1^ However, scars in the middle of the neck can create cosmetic disadvantages and patient discomfort.^2^ To avoid an incision in the middle of the neck, an endoscopic approach was first introduced in 1996.^3^ Since then, the endoscopic approach has been widely used to remove thyroid tumors. The endoscopic approach is highly dependent on surgeon skill, and the lack of flexibility of the two-dimensional operating screen and instruments can limit thyroid tumor removal.^4^, ^5^ However, a three-dimensional, expandable surgical field of view and the use of flexible surgical instruments for robotic thyroidectomy improved perioperative and operative outcomes and overcame the limitations of an endoscopic approach.^6^

Transaxillary and Bilateral Axillo-Breast Approach Robotic Thyroidectomy (BABART) techniques are widely used.^7^ BABART was first introduced in 2008.^8^ BABART has been compared with the bilateral axillo-breast approach endoscopic thyroidectomy (BABAET)^9^ and to conventional OT.^10^, ^11^, ^12^ However, evidence of the surgical safety and completeness of BABART is insufficient.

On the other hand, Transoral Robotic Thyroidectomy (TORT) requires less extensive dissection to create working space compared to BABART. The oral mucosal incision site of TORT can result in better healing.^13^ However, the effectiveness of TORT in comparison with BABART has yet to be fully evaluated.^14^

Therefore, the purpose of our meta-analysis is to evaluate the safety and effectiveness of BABART by comparing it with TORT, conventional OT, and BABAET in terms of surgical completeness, perioperative outcomes, and postoperative complications. To the best of our knowledge, this is the first meta-analysis to directly compare BABART to other approaches.

## Methods

### Search strategy

**Figure 1:**
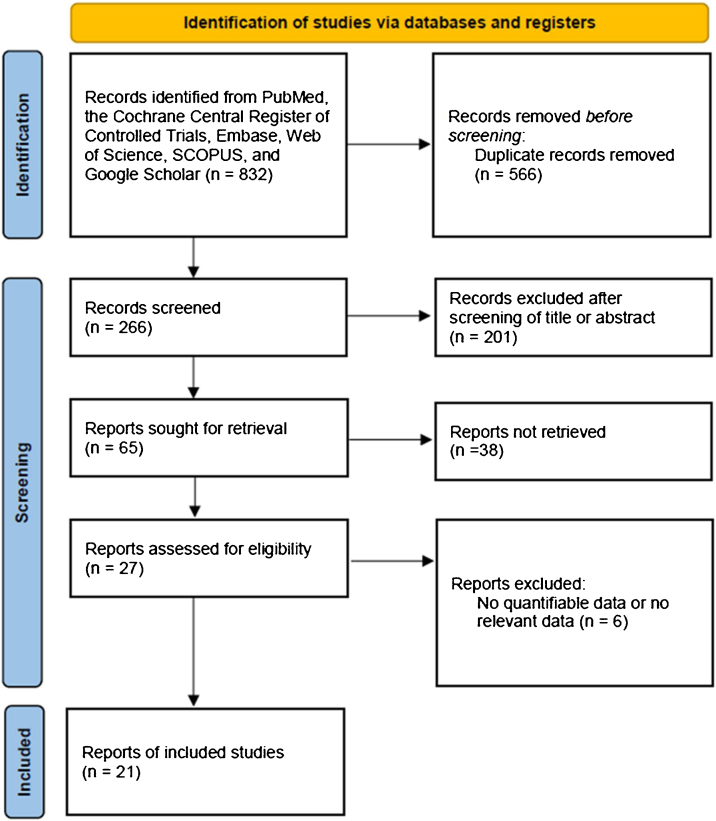
Diagram of study selection.

We searched for and collected papers published before October 2023 from PubMed, SCOPUS, Embase, Web of Science, Google Scholar, and Cochrane databases. Retrospective or prospective studies written only in English were included. “Thyroidectomy, transoral thyroidectomy, remote-access thyroid surgery, minimally invasive surgery, robotic thyroidectomy, robotic thyroid surgery, surgical approaches, bilateral axillo-breast approach thyroidectomy, bilateral axillo-breast approach, open thyroidectomy, thyroid neoplasm, thyroid carcinoma, thyroid nodule, cosmesis, and comparison” were used as the search terms. Among the searched studies, the two authors excluded those that did not mention BABART in the title and abstract. The selected studies were determined for inclusion in the meta-analysis by full text reading. A flow-chart for selecting eligible studies is summarized in Fig. 1. We registered the study protocol in the Open Science Framework (https://osf.io/kz9se/). This study was conducted with the Preferred Reporting Items for Systematic Review and Meta-Analysis Protocols 2020 statement.

### Data extraction and risk of bias assessment

From the included studies, number of patients, drainage amount, cosmetic satisfaction scores, operation time, days of hospitalization after operation, postoperative pain scores, retrieved Lymph Node (LN) number, postoperative thyroglobulin level, chyle leakage, incidental parathyroidectomy, postoperative infection, postoperative bleeding, transient or permanent postoperative hypoparathyroidism, transient or permanent Vocal Cord Palsy (VCP), and seroma were extracted. The data of the BABART group and other treatment group (conventional OT, BABAET, and TORT) were compared, and the p-values were extracted.^6^, ^11^, ^12^, ^14^, ^15^, ^16^, ^17^, ^18^, ^19^, ^20^, ^21^, ^22^, ^23^, ^24^, ^25^, ^26^, ^27^, ^28^, ^29^, ^30^, ^31^, ^32^ The data were organized in a standardized format.^33^, ^34^ The Newcastle-Ottawa Scale was used to evaluate non-randomized control studies.^35^

### Statistical analysis

We performed meta-analysis using 'R' statistical software (R Foundation for Statistical Computing, Vienna, Austria). When the original data were continuous, Mean Difference (MD) or Standard Mean Difference (SMD) were used for comparative analysis. For non-continuous variables, comparative analysis was performed using Odds Ratio (OR). In addition, we performed a sensitivity analysis to account for the impact of the included studies.

## Results

**Figure 2:**
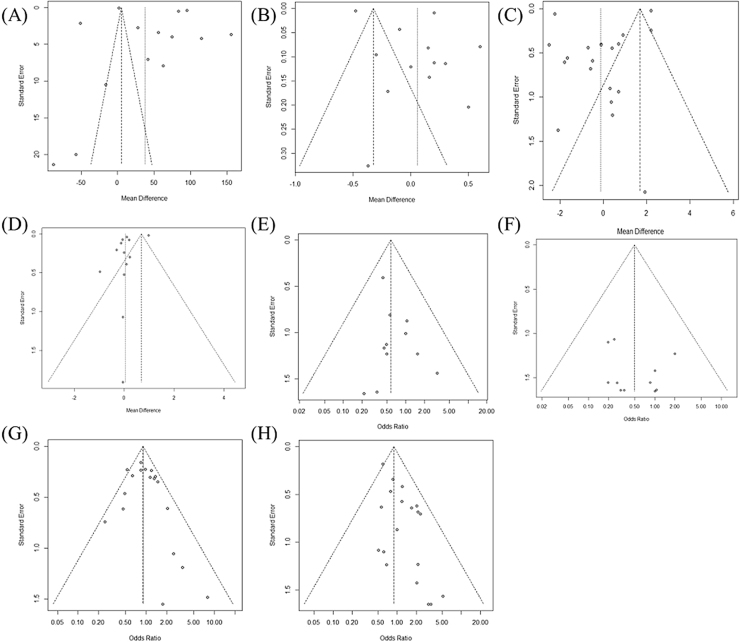
Funnel plot for publication bias of operation time (A), hospitalization (B), retrieved LN number (C), postoperative thyroglobulin level (D) or incidence of permanent hypoparathyroidism (E), permanent vocal cord palsy (F), transient hypoparathyroidism (G), and transient vocal cord palsy (H).

We analyzed data from 8830 patients included in 22 studies. The characteristics of each study are summarized in Table 1. In Fig. 2, Egger's test and Begg's funnel plot analysis confirmed that there was no publication bias in the included studies as follows: operation time (0.1032), hospitalization (0.3126), retrieved LN number (0.1302), postoperative thyroglobulin level (0.5011) or incidence of permanent hypoparathyroidism (0.1121), permanent VCP (0.5193), and transient hypoparathyroidism (0.2093). However, the extraction results for drainage amount; cosmetic satisfaction; postoperative pain score; and incidence of chyle leak, hematoma, infection, and incidental parathyroidectomy were insufficient to perform a funnel plot or advanced regression-based assessment. Therefore, publication bias could not be assessed. Transient VCP suggested the possibility of bias from the results of Egger's test and Begg's funnel plot (p <  0.001). Duval and Tweedie’s trim and fill results confirmed no significant difference between the observed and adjusted values (0.8069 [0.6474; 1.0057] vs. 0.79007 [0.5917; 1.001]). Therefore, the studies included in this meta-analysis can be stated to be unbiased in nature.

**Table 1:** The characteristics of the included studies.

Study	Design	Total number of patients (n)	Age of patients with robotic approach (years, mean ± SD)	Sex (F/M)	Nation	Body mass index (kg/m^2^, mean ± SD)	Tumor size (cm, mean ± SD or median [range])	Pathology	Comparison	Total number of patients (Conparison)	Control group	Total number of patients (Control group)	Outcomes
Kim 2011	Retrospective	302	41.3 ± 7.8	260/42	Korea	22.7 ± 2.7	0.7 ± 0.2	PTC (less than 1 cm)	BABART	69	Conventional OT	138	Drainage amount, hospital days, retrieved LN number, operation time, postoperative thyroid hormone level, incidence of chyle leakage, hematoma, wound infection, permanent & transient hypocalcemia, permanent & transient VCP
Lee 2011	Retrospective	216	43.7 ± 7.4	182/20	Korea	23.4 ± 3.0	0.82 ± 0.38	PTC (less than 1 cm)	BABART	108	Conventional OT	108	Postoperative thyroid hormone level
Tae 2012	Retrospective	218	38.9 ± 10.6	202/16	Korea	24.8 ± 4.3	2.24 ± 0.99	Benign or malignant thyroid tumor	BABART	113	BABAET	105	Drainage amount, retrieved LN number, operation time, cosmetic satisfaction (3mo), incidence of hematoma, permanent & transient hypocalcemia, permanent & transient VCP
Kim 2015	Retrospective	600	39.5 ± 0.5	532/68	Korea	NA	0.60 ± 0.03	Well-differentiated thyroid cancers	BABART	300	Conventional OT	300	Hospital days, retrieved LN number, operation time, postoperative thyroid hormone level, incidence of chyle leakage, hematoma, wound infection, permanent & transient hypocalcemia, permanent & transient VCP
Kim 2014	Retrospective	515	38.9 ± 10.1	421/94	Korea	22.7 ± 3.5	0.78 ± 0.44	PTC or suspected PTC	BABART	123	Conventional OT	392	Retrieved LN numbers, postoperative thyroid hormone level, incidence of permanent & transient hypocalcemia, permanent & transient VCP
Kwak 2014	Retrospective	840	40.01 ± 9.8	697/143	Korea	23.14 ± 3.44	0.95 ± 0.76	PTC or suspected PTC	BABART	206	Conventional OT	634	Hospital days, retrieved LN numbers, operation time, indicence of hematoma, wound infection, permanent & transient hypocalcemia, transient VCP
Chai 2016	Prospective	54	38.9 ± 7.5	54/0	Korea	23.9 ± 4.0	0.9 ± 0.5	PTC	BABART	27	Conventional OT	27	Hospital days, retrieved LN numbers, operation time, cosmetic satisfaction, postoperative pain score, permanent & transient hypocalcemia, permanent & transient VCP
Cho 2016	Retrospective	218	40.81 ± 10.84	185/33	Korea	23.73 (17.58–33.37)	0.7 (0.2–2.5)	PTC	BABART	109	Conventional OT	109	Hospital stay, retrieved LN numbers, postoperative thyroid hormone level, incidence of chyle leakage, hematoma, wound infection, permanent & transient hypocalcemia, permanent & transient VCP
He 2016	Prospective	100	40.9 ± 9.8	83/17	China	24.9 ± 3.5	50.7 ± 33	PTC	BABART	50	Conventional OT	50	Drainage amount, retreived LN numbers, operation time, cosmetic satisfaction, postoperative pain score, postoperative thyroid hormone level, incidence of chyle leakage, hematoma, wound infection, transient hypocalcemia, transient VCP
Kim 2016	Retrospective	229	38.9 ± 0.9	212/17	Korea	NA	0.50 ± 0.05	PTC	BABART	112	Conventional OT	117	Hospital days, retrieved LN numbers, operation time, cosmetic satisfaction, postoperative pain score, postoperative thyroid hormone level, incidence of chyle leakage, hematoma, wound infection, permanent & transient hypocalcemia, permanent & transient VCP
Paek 2016	Prospective	29	37.0 ± 10.4	25/4	Korea	NA	0.81 ± 0.62	Well-differentiated thyroid cancers	BABART	15	Conventional OT	14	Operation time, postoperative pain score
Chai 2017	Retrospective	86	30.8 ± 12.0	71/15	Korea	21.0 ± 2.8	2.8 ± 0.6	Thyroid cancer (larger than 2 cm)	BABART	21	Conventional OT	65	Hospital days, retrieved LN numbers, postoperative thyroid hormone level, permanent & transient hypocalcemia, permanent & transient VCP
Chai 2018	Retrospective	100	41.2 ± 9.4	93/7	Korea	22.8 ± 62.6	1.1 ± 0.8	PTC, benign, or follicular neoplasm	BABART	50	TORT	50	Hospital days, retrieved LN numbers, operation time, postoperative pain score (3day), incidence of wound infection, permanent & transient hypocalcemia, permanent & transient VCP
Kim 2017	Retrospective	578	39.6 ± 7.5	574/4	Korea	22.2 ± 2.7	0.6 ± 0.3	PTC	BABART	289	BABAET	289	Drainage amount, hospital days, retrieved LN numbers, operation time, postoperative thyroid hormone level, incidence of chyle leakage, hematoma, permanent & transient hypocalcemia, permanent & transient VCP
Kim 2018	Prospective	90	39.8 ± 10.7	83/7	Korea	NA	0.9 ± 0.5	PTC, benign, or follicular neoplasm	BABART	43	TORT	47	Hospital days, retrieved LN numbers, operation time, cosmetic satisfaction, postoperative thyroid hormone level, incidence of chyle leakage, hematoma, permanent & transient hypocalcemia, permanent & transient VCP
Paek 2018	Retrospective	376	36.4 ± 9.0	297/79	Korea	NA	0.83 ± 0.64	PTC	BABART	71	Conventional OT	305	Retrieved LN numbers, postoperative thyroid hormone level, incidence of chyle leakage, wound infection, permanent & transient hypocalcemia, permanent & transient VCP
Bae 2019	Retrospective	369	41.12 ± 8.97	348/21	Korea	22.99 ± 3.23	0.78 ± 0.43	Differentiated thyroid cancer	BABART	123	Conventional OT	246	Retrieved LN numbers, operation time, postoperative thyroid hormone level, incidence of chyle leakage, hematoma, wound Infection, permanent & transient hypocalcemia, permanent & transient VCP
Chae 2020	Retrospective	70	40.88 ± 9.80	9/61	Korea	23.60 ± 4.31	0.75 ± 0.35	PTC or follicular variant of PTC	BABART	56	TORT	14	Hospital days, retrieved LN number, postoperative pain score, incidence of wound infection, transient hypocalcemia, transient VCP
Choi 2020	Retrospective	1210	37.9 ± 10.02	1171/39	Korea	NA	1.45 ± 1.07	Benign, follicular (<5 cm), or malignant thyroid tumor	BABART	605	BABAET	605	Operation time, hospital days, retrieved LN numbers, postoperative thyroid hormone level, incidence of wound infection, permanent hypocalcemia, permanent VCP
He 2022	Prospective	99	44.6 ± 11.8	21/78	China	25.2 ± 14.2	0.35 ± 0.33	PTC	BABART	50	TORT	49	Drainage amount, retrieved LN numbers, operation time, cosmetic satisfaction, postoperative pain score, incidence of wound infection
Yang 2022	Retrospective	564	40.03 ± 9.7	492/72	Korea	23.27 ± 3.81	0.96 ± 0.95	Benign or malignant thyroid tumor	BABART	316	TORT	248	Hospital days, retrieved LN numbers, postoperative pain score, incidence of chyle leakage, hematoma, wound infection, permanent & transient hypocalcemia, permanent & transient VCP
Zhang 2023	Retrospective	757	31.25 ± 2.07	680/72	China	21.88 ± 0.62	6.3 ± 4.4	Benign or follicular (<5 cm), or malignant thyroid nodules at low risk	BABART	393	BABAET	364	Operation time, hospital days, drainage amount, retrieved LN numbers, incidence of permanent & transient hypocalcemia, permanent & transient VCP

NA, Not Available; OT, Open Thyroidectomy; PTC, Papillary Thyroid Cancer; BABART, Bilateral Axillo-Breast Approach Robotic Thyroidectomy; BABAET, Bilateral Axillo-Breast Approach Endoscopic Thyroidectomy Endoscopic Approach; TORT, Transoral Robotic Thyroidectomy; LN, Lymph Node; VCP, Vocal Cord Palsy.

### Operation- and postoperative course-related measurements between treatment and conventional OT

**Figure 3:**
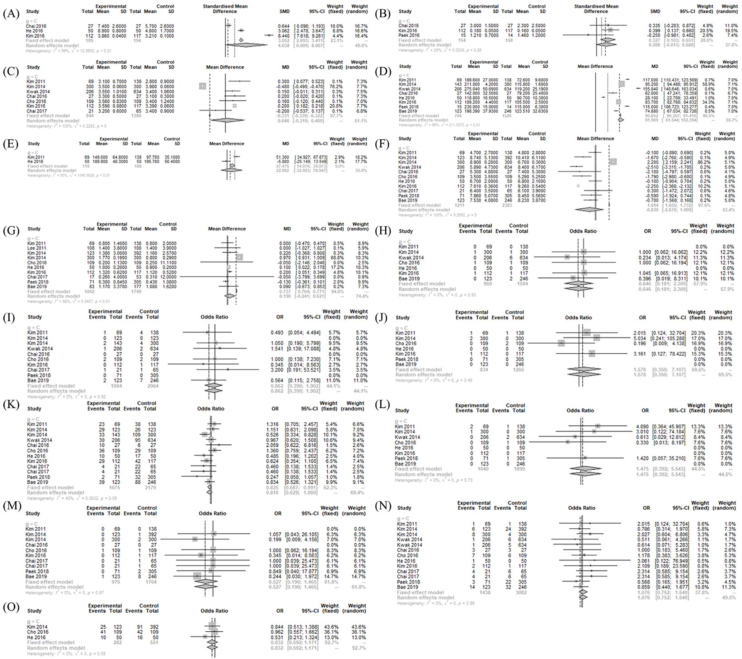
Operation- and postoperative course-related measurements between BABART and conventional thyroidectomy. Forest plot of standard mean difference of cosmetic satisfaction (A), postoperative pain score (B), hospital days (C), operation time (D), drainage amount (E), retrieved lymph node number (F), and postoperative thyroid hormone level (G), odd ratios of complications (hematoma (H), chyle leakage (I), permanent hypoparathyroidism (J), transient hypoparathyroidism (K), postoperative infection (L), permanent vocal cord palsy (M), transient vocal cord palsy (N), and incidental parathyroidectomy (O)) (total: number of participants per group). SD, Standard Deviation; SMD, Standardized Mean Difference; MD, Mean Difference; CI, Confidence Interval; C, Conventional open thyroidectomy.

The operative time (MD = 91.969 [81.544; 102.394]; I^2^ = 99%), postoperative pain score (SMD = 0.327 [0.103; 0.551]; I^2^ = 25%), and postoperative cosmetic satisfaction score (SMD = 4.038 [0.009; 8.067]; I^2^ = 99%) were significantly larger in the BABART group compared to conventional OT group. There was no significant difference in number of retrieved LN (MD = −0.830 [−2.670; 1.009]; I^2^ = 100%), drainage amount (MD = 22.992 [−32.963; 78.947]; I^2^ = 95%), hospitalization periods (MD = 0.0463 [−0.3163; 0.4090]; I^2^ = 99.9%), and postoperative thyroglobulin level (MD = 0.0190 [−0.241; 0.621]; I^2^ = 98%) (Fig. 3).

In postoperative morbidities, there was no significant difference in chyle leakage (OR = 0.862 [0.390; 1.902]; I^2^ = 0%), hematoma (OR = 0.646 [0.181; 2.309]; I^2^ = 0%), incidental parathyroidectomy (OR = 0.832 [0.592; 1.171]; I^2^ = 0%), infection (OR = 1.475 [0.392; 5.543]; I^2^ = 0%), transient VCP (OR = 1.076 [0.752; 1.540]; I^2^ = 0%), permanent VCP (OR = 0.527 [0.190; 1.465]; I^2^ = 0%), and permanent hypoparathyroidism (OR = 1.578 [0.350; 7.107]; I^2^ = 0%). However, transient hypoparathyroidism (OR = 0.825 [0.687; 0.991]; I^2^ = 43%) was less frequent in the BABART group compared to conventional OT group (Fig. 3).

### Operation- and postoperative course-related measurements between treatment and BABAET

**Figure 4:**
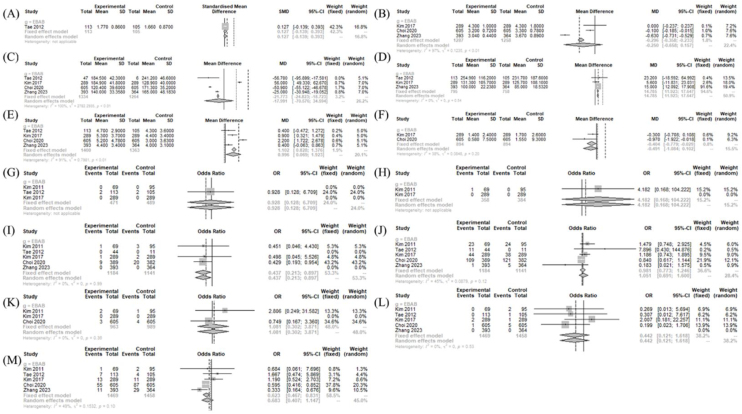
Operation- and postoperative course-related measurements between BABART and BABAET. Forest plot of standard mean difference of cosmetic satisfaction (A), hospital days (B), operation time (C), drainage amount (D), and retrieved lymph node number (E), and postoperative thyroid hormone level (F), odd ratios of complications (hematoma (G), chyle leakage (H), permanent hypoparathyroidism (I), transient hypoparathyroidism (J), postoperative infection (K), permanent vocal cord palsy (L), and transient vocal cord palsy (M)) (total: number of participants per group). SD, Standard Deviation,; SMD, Standardized Mean Difference; MD, Mean Difference; CI, Confidence Interval; EBAB, Bilateral Axillo-Breast Approach endoscopic thyroidectomy endoscopic approach.

The drainage amount (MD = 14.785 [11.923; 17.647]; I^2^ = 0%) and retrieved LN numbers (MD = 0.996 [0.069; 1.923]; I^2^ = 91%) was significantly larger in the BABART group compared to BABAET group. The postoperative thyroglobulin level (MD = −0.404 [−0.779; −0.029]; I^2^ = 38%) was significantly lower in the BABART group compared to BABAET group. There was no significant difference in postoperative cosmetic satisfaction score (SMD = 0.127 [−0.139; 0.393]), hospitalization periods (MD = −0.2503 [−0.6577; 0.1572]; I^2^ = 97.1%), and operative time (MD = −17.991 [−70.576; 34.594]; I^2^ = 100%) between BABART and BABAET group (Fig. 4).

In postoperative morbidities, there was no significant difference in chyle leakage (OR = 4.182 [0.168; 104.222]), hematoma (OR = 0.928 [0.128; 6.709]), infection (OR = 1.081 [0.302; 3.871]; I^2^ = 0%), permanent VCP (OR = 0.442 [0.121; 1.618]; I^2^ = 0%), and transient hypoparathyroidism (OR = 0.981 [0.773; 1.246]; I^2^ = 45%). However, transient VCP (OR = 0.623 [0.467; 0.831]; I^2^ = 49%) and permanent hypoparathyroidism (OR = 0.437 [0.213; 0.897]; I^2^ = 0%) were less frequent in the BABART group compared to BABAET group (Fig. 4).

### Operation- and postoperative course-related measurements between treatment and TORT

**Figure 5:**
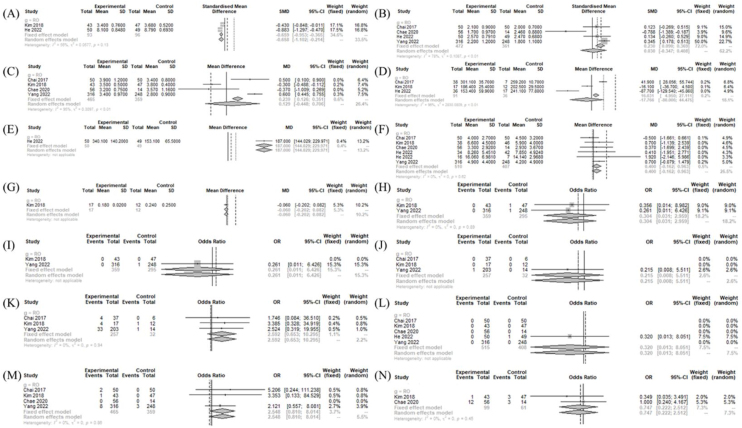
Operation- and postoperative course-related measurements between BABART and TORT. Forest plot of standard mean difference of cosmetic satisfaction (A), postoperative pain score (B), hospital days (C), operation time (D), drainage amount (E), retrieved lymph node number (F), and postoperative thyroid hormone level (G), odd ratios of complications (hematoma (H), chyle leakage (I), permanent hypoparathyroidism (J), transient hypoparathyroidism (K), postoperative infection (L), transient vocal cord palsy (M), and incidental parathyroidectomy (N)) (total: number of participants per group). SD, Standard Deviation; SMD, Standardized Mean Difference; MD, Mean Difference; CI, Confidence Interval; RO, Transoral Robotic Thyroidectomy.

The drainage amount (MD = 187.000 [144.029; 229.971]) was significantly larger in the BABART group compared to TORT group. The postoperative cosmetic satisfaction score (SMD = −0.658 [−1.102; −0.214]; I^2^ = 56%) was significantly lower in the BABART group compared to TORT group. There was no significant difference in operative time (MD = −17.766 [−80.006; 44.475]; I^2^ = 96%), hospitalization periods (MD = 0.1292 [−0.4475; 0.7058]; I^2^ = 94.8%), number of retrieved LN (MD = 0.400 [−0.162; 0.963]; I^2^ = 0%), pain score (SMD = 0.030 [−0.347; 0.408]; I^2^ = 78%), and postoperative thyroglobulin level (MD = -0.060 [−0.202; 0.082]) (Fig. 5).

In postoperative morbidities, there was no significant difference in chyle leakage (OR = 0.261 [0.011; 6.426]), hematoma (OR = 0.304 [0.031; 2.959]; I^2^ = 0%), incidental parathyroidectomy (OR = 0.747 [0.222; 2.512]; I^2^ = 0%), infection (OR = 0.320 [0.013; 8.051]), transient VCP (OR = 2.548 [0.810; 8.014]; I^2^ = 0%), transient hypoparathyroidism (OR = 2.592 [0.653; 10.295]; I^2^ = 0%), and permanent hypoparathyroidism (OR = 0.215 [0.008; 5.511]) between BABART group and TORT group (Fig. 5).

### Comparison of operation and postoperative course-related measurements according to operation type

Compared to conventional OT, the BABART group showed longer operative time (MD = 77.1700 [33.5309; 120.8092], p <  0.0001), and greater patient cosmetic satisfaction (SMD = 4.0379 [0.0085; 8.0672], p = 0.0016) with statistical significance. There were no significant differences in other operation-related measurements between the two groups (Table 2).

**Table 2:** Comparison of operation-related measurements according to operation type.

Comparison	Cosmetic satisfaction (SMD [95% CIs]; I^2^)	Postoperative pain score (SMD [95% CIs]; I^2^)	Hospitalization day (MD [95% CIs]; I^2^)	Operation time (MD [95% CIs]; I^2^)	Drainage amount (MD [95% CIs]; I^2^)	Retrieved lymph node number (MD [95% CIs]; I^2^)	Postoperative thyroid hormone level (MD [95% CIs]; I^2^)
Conventional OT	n = 3	n = 3	n = 7	n = 8	n = 2	n = 11	n = 10
	4.0379 [0.0085; 8.0672]; 99.2%	0.3268 [0.1028; 0.5508]; 25.2%	0.0463 [−0.3163; 0.4090]; 99.9%	91.969 [81.544; 102.394]; 99%	22.9924 [−32.9626; 78.9473]; 94.9%	−0.8302 [−2.6696; 1.0092]; 99.8%	0.1901 [−0.2405; 0.6207]; 98.2%
BABAET	n = 1	NA	n = 3	n = 4	n = 3	n = 4	n = 2
	0.1268 [−0.1392; 0.3927]; NA		−0.2503 [-0.6577; 0.1572]; 97.1%	−17.9908 [−70.5761; 34.5944]; 99.6%	14.7851 [11.9231; 17.6470]; 0.0%	0.9958 [0.0688; 1.9228]; 90.6%	−0.4039 [−0.7789; −0.0290]; 37.8%
TORT	n = 2	n = 4	n = 4	n = 3	n = 1	n = 6	n = 1
	−0.6578 [−1.1020; −0.2135]; 56.1%	0.0302 [−0.3471; 0.4076]; 77.5%	0.1292 [−0.4475; 0.7058]; 94.8%	−17.7656 [−80.0059; 44.4747]; 95.7%	187.0000 [144.0291; 229.9709]; NA	0.4004 [−0.1623; 0.9631]; 0.0%	−0.0600 [−0.2018; 0.0818]; NA
p-value	0.0016	0.2951	0.4564	<0.0001	<0.0001	0.2005	0.1896

SMD, Standardized Mean Difference;MD, Mean Difference; CI, Confidence Interval; OR, Odds Ratio; NA, Not Available; OT, Open Thyroidectomy; BABAET, Bilateral Axillo-Breast Approach Endoscopic Thyroidectomy Endoscopic Approach; TORT, Transoral Robotic Tyroidectomy.

Compared to BABAET, the BABART group showed greater postoperative drainage (MD = 14.7851 [11.9231; 17.6470], p < 0.0001) with statistical significance. The BABART group exhibited better surgical completeness as measured by the lower postoperative thyroglobulin level (MD = −0.4039 [−0.7789; -0.0290], p = 0.1896) and higher retrieved LN numbers (MD = 0.9958 [0.0688; 1.9228], p = 0.2005) than BABAET group. However, there were no statistical significance (Table 2).

BABART group induced greater postoperative drainage (MD = 187.0000 [144.0291; 229.9709], p < 0.0001) and showed lower patient cosmetic satisfaction than TORT group (SMD = −0.6578 [−1.1020; -0.2135] p = 0.0016). There were no significant differences in other operation-related measurements between the two groups (Table 2).

There were no significant differences in other postoperative morbidities between BABART group and other approaches (Table 3).

**Table 3:** Comparison of postoperative morbidities according to operation type.

Comparison	Hematoma (OR [95% CIs]; I^2^)	Chyle leakage (OR [95% CIs]; I^2^)	Permanent hypoparathyroidism (OR [95% CIs]; I^2^)	Transient hypoparathyroidism (OR [95% CIs]; I^2^)	Postoperative infection (OR [95% CIs]; I^2^)	Permanent vocal cord palsy (OR [95% CIs]; I^2^)	Transient vocal cord palsy (OR [95% CIs]; I^2^)
Conventional OT	n = 5	n = 4	n = 7	n = 12	n = 5	n = 8	n = 13
	0.6460 [0.1807; 2.3094]; 0.0%	0.862 [0.390; 1.902]; 0.0%	1.578 [0.350; 7.107]; 0.0%	0.8253 [0.6871; 0.9914]; 43.2%	1.4750 [0.3925; 5.5433]; 0.0%	0.5275 [0.1900; 1.4647]; 0.0%	1.0764 [0.7522; 1.5404]; 0.0%
BABAET	n = 1	n = 1	n = 3	n = 5	n = 2	n = 4	n = 5
	0.9279 [0.1283; 6.7088]; NA	4.1825 [0.1678; 104.2219]; NA	0.4367 [0.2125; 0.8974]; 0.0%	0.9812 [0.7727; 1.2460]; 45.3%	1.0807 [0.3017; 3.8705]; 0.0%	0.4418 [0.1206; 1.6182]; 0.0%	0.6230 [0.4672; 0.8309]; 48.8%
TORT	n = 2	n = 1	n = 1	n = 3	n = 1	NA	n = 3
	0.3044 [0.0313; 2.9587]; 0.0%	0.2607 [0.0106; 6.4263]; NA	0.2148 [0.0084; 5.5106]; NA	2.5920 [0.6526; 10.2951]; 0.0%	0.3201 [0.0127; 8.0506];NA		2.5483 [0.8103; 8.0143]; 0.0%
p-value	0.7637	0.4663	0.3834	0.1654	0.6880	0.8334	0.0089

OR, Odds Ratio; CI, Confidence Interval; NA, Not Available; OT, Open Thyroidectomy; BABAET, Bilateral Axillo-Breast Approach Endoscopic Thyroidectomy Endoscopic Approach; TORT, Transoral Robotic Thyroidectomy.

The English in this document has been checked by at least two professional editors, both native speakers of English. For a certificate, please see: http://www.eworldediting.com/pdf/examples/example_001.php?bo_table=c_request&wr_id=59611&t=D.

### Sensitivity analyses

Sensitivity analyses were performed to determine differences in integrated estimates in such a way that the meta-analysis was repeated excluding one study each time. All results were consistent with those above.

## Discussion

Bilateral axillo-breast approach on thyroid can provide surgeons a symmetric surgical view of the thyroid through a midline approach as in conventional OT but with the largest operating angle for surgical instruments.^36^ In addition, good cosmetic satisfaction of patients can be expected due to the smaller wounds.^36^ Therefore, BABART has been evaluated as safe and effective for thyroid surgery.^37^, ^38^ However, meta-analysis on the most recent data directly comparing BABART with other approaches remains insufficient.

In our study, BABART was not inferior to other thyroidectomy approaches in many respects but was inferior to TORT and BABAET in drainage amount. More extensive dissection is required to obtain the necessary operation fields in BABART, resulting in a significantly larger drainage amount. A previous study showed that the cumulative drainage volume of BABART was larger than that of conventional OT.^15^

For dissection time, BABART produced significantly longer operation times than the conventional OT in our study. This discrepancy from previous studies is potentially due to the extended time for flap dissection and docking of robotic instruments in BABART.^20^, ^24^, ^39^ If the surgeon's experience with BABART increases, the operation time may decrease, but the difference from OT operation time may not decrease significantly.^36^ However, an increase in the number of robotic surgeries may help overcome longer operation times.^39^

However, hospitalization and postoperative pain scores did not significantly differ between other approaches and BABART. BABART reported decreased hospitalization period compared to BABAET. The average length of hospitalization after BABART was 3–5 days, and there was no significant difference between conventional OT and the length of stay in previous reports.^19^, ^20^ The postoperative pain scores were different in each previous study,^12^, ^21^ but BABART was reported to be at least equivalent to conventional OT.^36^

In addition, the cosmetic satisfaction score of BABART was significantly higher than that of conventional OT but significantly lower than that of TORT. Even though the measurement scale for cosmetic satisfaction was different in previous studies, the cosmetic satisfaction of BABART was significantly higher compared to conventional OT.^19^, ^20^, ^40^ TORT has better cosmetic outcomes than BABART because wounds in the oral mucosa heal well and are not readily visible.^14^

For operative outcomes, retrieved LN number and postoperative thyroglobulin level were not significantly different between other approaches and BABART. However, in the BABART group, retrieved LN number was significantly greater and postoperative thyroglobulin level was significantly lower than those of BABAET. The appropriateness of oncologic control can be based on the retrieved LN number from central node dissection.^36^ Retrieved central LN number previously was significantly lower with BABART compared to OT,^21^ but there was no significant difference in our results. BABAET may have limitations in central LN number because the instruments are linear and access to the peritracheal area or upper mediastinum is limited.^14^ A robotic system was recommended to solve this limitation of BABAET,^27^ and our results potentially support this recommendation.

The surgical completeness of thyroidectomy can be evaluated through serum thyroglobulin level and total dose of radioactive iodine.^41^ The thyroglobulin level is an index that can determine the degree of remnant thyroid; a previous study showed no significant difference between BABART and OT.^39^ Choi et al. reported that the recurrence rate of thyroid malignancy was lower in BABART compared to BABAET.^6^

Regarding postoperative complications, BABART exhibited significantly better results in VCP or hypoparathyroidism than conventional OT or BABAET group. Other complications showed no significant difference between BABART and other approaches. Therefore, BABART did not appear to cause any additional risk compared to other approaches in terms of postoperative complications, especially in hypoparathyroidism and VCP.

This study has several limitations. First, included studies were mostly retrospective and randomized controlled trials, potentially creating selection bias. The subjects or conditions of included RCT may not be consistent. In addition, there may be publication bias or selection bias in the RCT itself. Second, the complication rate was not high in most studies, making it difficult to identify the exact difference. Third, complications such as chest numbness after BABART were not evaluated. Fourth, different evaluation scales for postoperative pain or cosmetic satisfaction may increase heterogeneity. Fifth, heterogeneity may be increased because postoperative management (pain control, wound care, or postoperative dieting) and instruments used for surgery differ by institution and surgeon's skill level or experience.

## Conclusion

BABART was inferior to TORT in drainage and cosmetic satisfaction. However, in terms of surgical completeness and transient VCP, BABART was better than BABAET. BABART also showed better results in lower chance of transient hypoparathyroidism compared to BABAET and conventional OT. Since BABART had higher cosmetic satisfaction than conventional OT and did not report additional complications, an individualized appropriate approach for patients with thyroid tumors should be selected.

## Conflicts of interest

The authors declare no conflicts of interest.
